# Symptomatic Atlas Hypoplasia in a Latin-American Patient: Case Report and Literature Review

**DOI:** 10.5435/JAAOSGlobal-D-21-00041

**Published:** 2021-05-04

**Authors:** Matias Pereira Duarte, Gasto Camino Willhuber, Matias Petracchi, Marcelo Gruenberg, Carlos Alberto Sola

**Affiliations:** From the Hospital Italiano de Buenos Aires, Spinal Pathology Service, Almagro, CABA, Argentina. Orthopedic Institute “Prof. Dr. Carlos E. Ottolengui”, Adult Spine Pathology Service, Hospital Italiano de Buenos Aires, CABA, Argentina.

## Abstract

**Background:**

Atlas hypoplasia is an infrequent cause of upper cervical stenosis. Only 24 cases in nonsyndromic adult population have been published. We are not aware of previous reports describing isolated fully formed atlas hypoplasia in a Latin-American patient. The purpose of this work was to report a case of an 80-year-old Argentinian woman with cervical myelopathy because of atlas hypoplasia and a literature review about this subject.

**Methods:**

A clinical case and an extended review of the literature are presented. We assessed from each case: age, sex, posterior atlanto-dens interval, surgical treatment, outcomes, and follow-up period.

**Results:**

Neurologic symptoms markedly improved after posterior decompression from severe to moderate in the Japanese Orthopaedic Association Scoring System and from four to three on the Nurick scale. Twenty-five patients were analyzed (mean 58.4 years, 32% female). The mean posterior atlanto-dens interval was 8.8 mm. Twenty-three patients underwent decompression alone, and two needed posterior fusion. All patients reported clinical improvement at an average follow-up of 13 months.

**Conclusion:**

Cervical myelopathy caused by fully formed atlas hypoplasia is not an exclusive pathology of far east population, and it may present in nonsyndromic patients. Surgical treatment by C1 laminectomy improved neurologic impairment.

**Study Design:**

Case report and literature review.

Cervical myelopathy in elderly patients occurs most frequently in the subaxial spine because of degenerative spondylosis.^1^ Spinal cord compression at the C1 level is infrequent because the cervical canal diameter is larger at this level.^2^ The available literature concerning upper cervical compression is based on anecdotal cases or small series.^3,4,5,6,7^ C1 stenosis etiology can be divided into congenital or acquired. Congenital pathologies correspond to any anatomical anomaly of the atlas, such as clefts or aplasias of anterior and posterior arches, os odontoideum, or dens hypertrophy, either alone or in combination.^8^ Isolated pure atlas hypoplasia (anatomically complete but small Atlas ring) is a very infrequent anomaly.^3^ A posterior midline cleft is the most usual encountered abnormality (3% to 4% in cervical radiographs), with a much higher prevalence than anterior arch defects (<1%).^4^ Many of these congenital anomalies, including atlas hypoplasia, may occur as part of different syndromes, such as Arnold-Chiari, Klippel-Feil, Down,^5^ and Turner syndrome; ankylosing spondylitis; achondroplasia (more often related to foramen magnum stenosis)^9^; and mucopolysaccharidosis, such as Morquio syndrome.^6,10,11^ On the other hand, acquired C1 stenosis may present as a consequence of trauma, osteoarthritis, or pannus formation because of evolved rheumatoid arthritis, which is unusual these days as a consequence of medical therapy improvements.

The objective of this study was to present a case of isolated symptomatic C1 hypoplasia in a Latin-American female patient. In addition, a literature review regarding this subject was done.

## Methods

After our Institutional Ethical Committee approval and patient informed consent, we present a clinical case and the data analysis from a narrative literature review. A bibliographic search was conducted according to the following criteria: articles in PUBMED including case reports and case series, involving humans aged 18 years or older, with C1 complete hypoplasia and with or without myelopathy. The search was achieved using the following MeSH terms: “C1 stenosis,” “atlas congenital anomalies,” and “cervical myelopathy.” We obtained additional cases from these articles' references.

Exclusion criteria included pediatric patients, with another anatomical anomaly such as medial cleft, total or partial agenesis, or any syndromic background, including Arnold-Chiari malformation, Klippel-Feil syndrome, gonadal dysgenesis, Down syndrome, Turner syndrome, ankylosing spondylitis, achondroplasia, Morquio syndrome, and ossification of posterior longitudinal ligament.

The following data were collected from each case if available: author, year of publication, patients' age, sex, posterior atlanto-dens interval (PADI, measured from posterior border of dens to the C1 lamina), surgical procedure, outcomes, follow-up period, and any other relevant information if necessary.

## Case Presentation

An 80-year-old woman without a relevant medical background reported a 6-year history of neck pain and gait disturbance. She also referred increased urinary frequency with occasional episodes of incontinence. The neurologic examination demonstrated hyperactive deep tendon reflexes in all four limbs with a positive bilateral Hoffmann sign. Lhermitte sign was positive, neither Babinski nor clonus signs were detected. The patient's gait was moderately spastic. Hand dexterity was progressively lost. The modified Japanese Orthopaedic Association Scoring System^12^ preoperatively scored 8 of 18 (severe impairment), and her Nurick scale was 4.^13^

**Figure 1:**
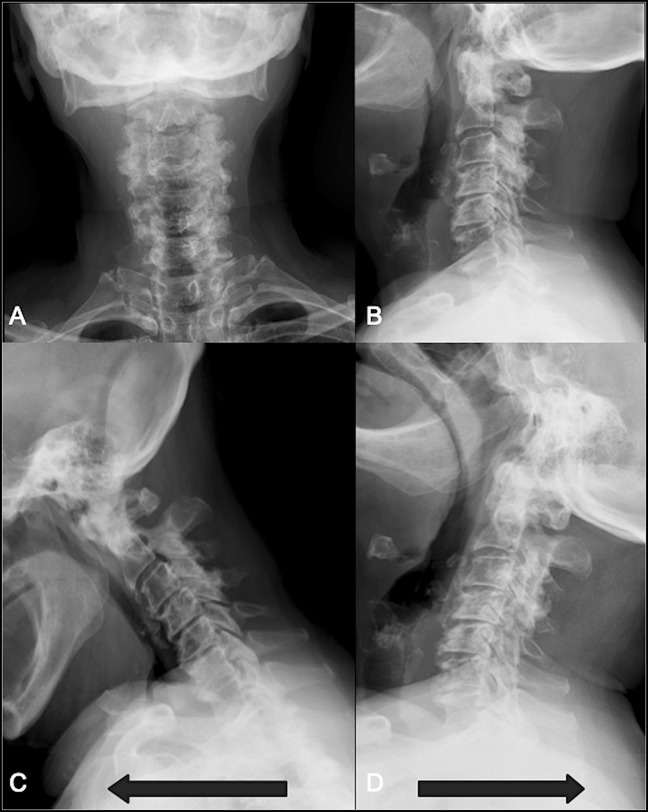
Anterior-posterior (**A**) and lateral (**B**) cervical radiographs showing a relatively small atlas. Flexion (**C**) and extension (**D**) cervical radiographs demonstrating no notable instability.

**Figure 2:**
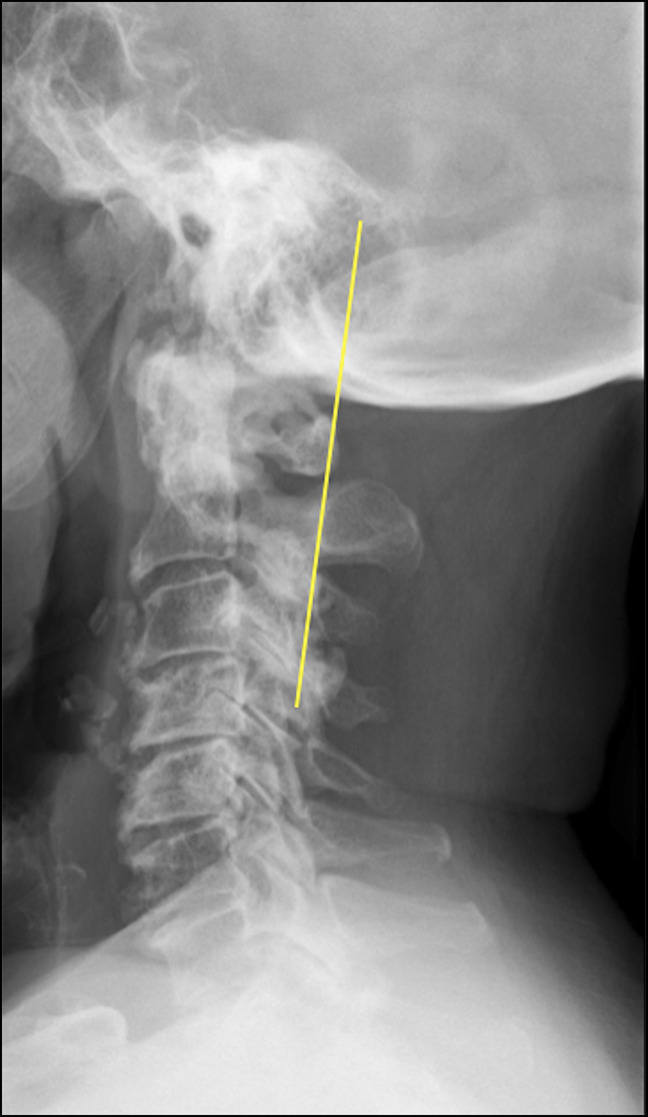
Lateral cervical radiographs showing the case. The C2/C3 spinolaminar line was marked, and the ventral aspect of the posterior arch of C1 lies ventral to this line. The C2/C3 spinolaminar line was drawn, starting from C3 and extending cranially through C2 to the lamina of C1. When the ventral lamina of C1 lay ventral to this line, the spinolaminar test is defined as positive, which indicated the possibility of the existence of a relatively narrow canal of C1.

**Figure 3:**
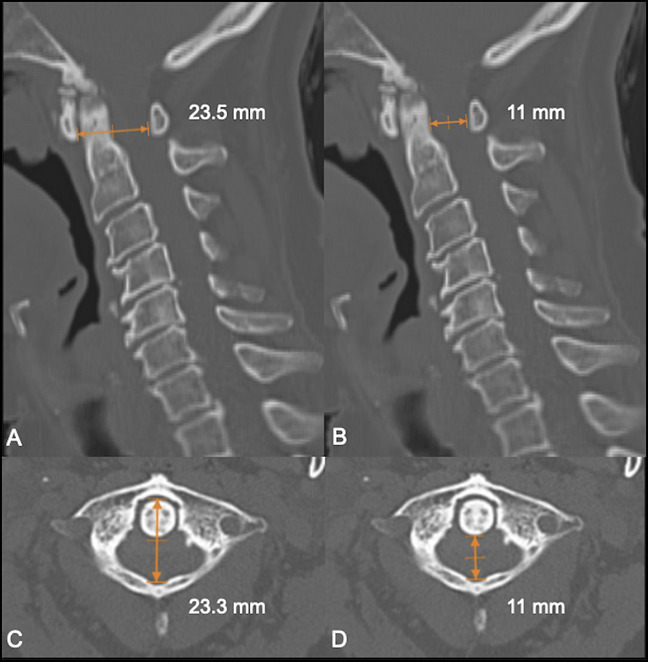
Sagittal (**A** and **B**) and axial (**C** and **D**) CT scan images showing decreased values of the C1 diameter and posterior atlanto-dens interval.

**Figure 4:**
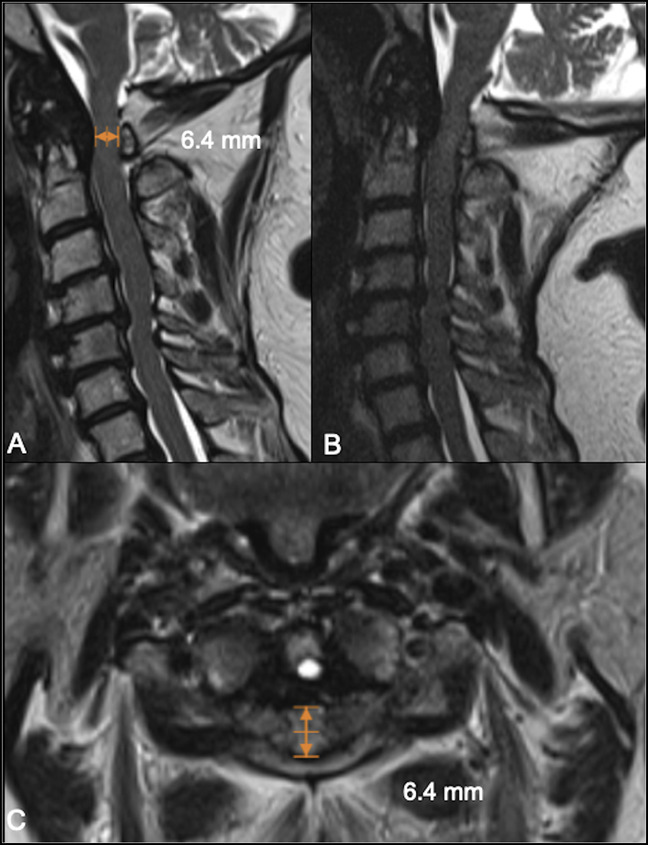
Sagittal (**A** and **B**) and axial (**C**) MRIs showing a minimal space available for the cord with values below 7 mm. There is a bone cyst at the tip of the dens and a thick transverse ligament that allows a narrow corridor for the medullary cord.

Lateral cervical spine radiographs revealed a small posterior arch of the atlas and bilateral ponticulus posticus (Figures 1, A and B), the C2/C3 spinolaminar line was marked, and the anterior aspect of the posterior arch of the atlas rested ventral to it (Figure 2). Spondylotic changes were present throughout the entire cervical spine. No atlantoaxial instability was detected on flexion and extension views (Figures 1C and D). Axial and sagittal CT scan revealed a hypoplastic intact posterior arch of the atlas. PADI measurement was 11 mm (Figure 3B and D). Magnetic resonance images revealed a remarkably flattened spinal cord at C1 and also a diffuse and subtle high signal image in the spinal cord on the STIR sequence suggesting edema because of compression. A hypertrophic transverse ligament and an odontoid bone cyst were also identified. The available space for the spinal cord, measured from the most dorsal aspect of the transverse ligament to the C1 lamina confluence, was narrowed to 6.5 mm (Figures 4A–C). As no instability signs were detected, the patient underwent decompression surgery by removing the posterior arch of the atlas without perioperative complications. This resulted in a notable improvement in limbs numbness and gait disturbance. At 12 months of follow-up, the postoperative Japanese Orthopaedic Association Scoring System score was 13 of 18 (moderate impairment), and Nurick grade was 3 with notable gait and numbness improvement.

## Case Series Analysis Results

Twenty-five cases were included in this review (Table 1), including the one presented in this article. All cases but eight corresponded to oriental ethnicity (Japanese and Chinese), two patients were from Belgium,^18^ four from India,^6^ and one from Saudi Arabia.^27^ This is the first report of a Latin-American female patient in America. Eight patients were female (32%). The average age was 58.4 years (ranging from 22 to 81). The mean PADI was 8.8 mm (ranging from 5.5 to 14 mm); for two patients, this value was not available (cases 17 and 20).^24,27^

**Table 1:** Literature Review of Fully Formed Atlas Hypoplasia Cases

Author (yr)	Case Number	Sex	Age	PADI (mm)	Treatment	Outcomes	Follow-up (mo)	Associated Characteristics
Ishii et al^14^ (1984)	1	M	58	8	C1 laminectomy	Residual moderate disability	NA	
Sawada et al^15^ (1989)	2	M	38	7	C1 laminectomy	Clinical improvement	NA	
Komatsu et al. ^[16]^ (1993)	3	M	56	7.7	C1 laminectomy + craniectomy	Clinical improvement	2	
Tokiyoshi et al^17^ (1994)	4	M	55	7	C1 laminectomy + craniectomy	Clinical improvement	12	
Benitah et al^18^ (1994)	5	F	41	7	C1 and C2 laminectomy	Clinical improvement	NA	Associated congenital hypertrophy of the laminae of C1 and C2
	6	M	78	8	C1 and C2 laminectomy	Clinical improvement	NA	Extensive unilateral osteophytes on the left C1–C2 joint in a violinist
Noguchi et al^19^ (1998)	7	M	81	10	C1 laminectomy	Residual moderate disability	NA	
Okamoto et al^20^ (1998)	8	NA	77	11	C1 laminectomy + posterior fusion	Good recovery	NA	
Phan et al^21^ (1998)	9	M	80	8	C1 laminectomy + craniectomy	Some improvement of his clinical symptoms	6	
	10	M	75	7	C1 laminectomy	Improvement of his clinical symptoms	2.5	
May et al^22^ (2001)	11	M	66	10	C1 laminectomy + craniectomy	Residual moderate disability	NA	
Nishikawa et al^23^ (2001)	12	M	82	12	C1 laminectomy	Clinical improvement	NA	
	13	M	72	11	C1 laminectomy	Clinical improvement	3	Died at 3 months because of dissecting abdominal aneurysm
	14	F	22	9	C1 laminectomy	Clinical improvement	72	
Tsuruta et al^7^ (2003)	15	F	79	8	C1 laminectomy	Clinical improvement—able to walk with a cane	18	Partial ossification of the transverse ligament
Chang et al^24^ (2007)	16	M	79	6.9	C1 laminectomy	Clinical improvement JOA from 3 to 11/17	NA	
	17	M	62	NA	C1 laminectomy + occipito-atlanto-axial fusion with wire fixation	Clinical improvement JOA from 13 to 17/17	NA	Atlantoaxial instability
Hsu et al^25^ (2007)	18	M	38	12.4	C1 laminectomy + duraplasty	Clinical improvement	NA	
Tang et al^26^ (2010)	19	F	58	5.5	C1 laminectomy	Notable improvement	NA	Partial ossification of the transverse ligament, hypertrophy of the dens
Bokhari and Baeesa^27^ (2012)	20	F	68	NA	C1 laminectomy	Clinical improvement	6	Partial ossification of the transverse ligament
Nehete et al^6^ (2018)	21	F	47	11	C1 laminectomy	Reduced neck pain, able to write	3	
	22	F	52	7	C1 laminectomy	Able to walk	18	
	23	M	60	14	C1 laminectomy	Spasticity better	12	Previously operated C2–C7 laminectomy
	24	M	35	5.5	C1 and C2 laminectomy	Minimal improvement	12	
Pereira duarte et al	25	F	80	11	C1 laminectomy	Clinical improvement JOAm from 8 to 13/18 Nurick from 4 to 3.	12	

JOAm = Japanese Orthopaedic Association Scoring System, PADI = posterior atlanto-dens interval

Twenty-three patients underwent decompression surgery with their variants: 15 patients, C1 laminectomies alone (60%); 3 patients, C1 and C2 laminectomy (cases 5, 6, and 24)^6,18^; 4 patients required C1 laminectomy associated with craniectomy (cases 3, 4, 9, and 11)^16,17,21,22^; and 1 patient was treated with C1 laminectomy and duraplasty (case 18).^25^ Two of 25 patients were treated by C1 laminectomy and posterior fusion; case 17 was due to radiographic atlantoaxial instability^24^; and in case 8,^20^ this information was not available. None of the articles reviewed assessed technical surgical problems or intraoperative complications related to vascular malformations or neurological anomalies associated.

Outcomes measurements were heterogeneous, the overall analysis showed consistency in clinical improvement after decompression; however, only two cases reported by Chan et al^24^ were evaluated with de JOA score preoperatively and postoperatively. Follow-up data were only available in 13 cases (52%), and the average period of follow-up was 13 months (ranging from 2 to 72 months).

## Discussion

We report a case of one myelopathic Latin-American female patient with C1 stenosis because of atlas hypoplasia and a thorough literature review in nonsyndromic adult patients. As far as we know, we present the largest literature review of patients affected with this pathology, and this is the first case reported in America. We are not aware of previous reports in this region, this could be due to unfamiliarity to this entity from clinicians, radiologists, or surgeons.

Wackenheim^28^ first described hypoplasia of the atlas in 1974 as a cause of upper cervical stenosis without myelopathy. Ishii et al^14^ and Sawada et al^15^ were the next in describing cases of atlas hypoplasia accompanied by cervical myelopathy in nonsyndromic adult patients. Throughout the years, different authors^3,6,7,23,24^ have published their cases along with literature reviews. As far as we concern, all of them are heterogeneous—including adult and pediatric patients, with and without syndromic backgrounds—and incomplete series of patients because many cases have never been recorded in any of them.^24-26^ Some cases^14,19,20^ were reported in low impact far east journals, and they were retrieved from Tsuruta et al^7^ report.

After revising each paper meticulously, 28 cases were excluded: 16 pediatric patients^3,6,25^ (some of them with congenital syndromes), 1 subaxial multilevel stenosis, 1 Arnold-Chiari malformation,^7^ 1 Klippel-Feil syndrome, 1 ankylosing spondylitis, 1 Morquio syndrome,^6^ 1 patient with a complete absence of the posterior arch (Currarino classification type E),^29^ 3 retroodontoideal mass of uncertain origin,^7,30^, 2 patients with ossification of posterior longitudinal ligament,^7,24^ and 1 adult with bipartite atlas and atlantoaxial instability that refused surgery.^3,31^ A total of 25 patients were reviewed along with ours.

### Anatomy and Embryology

To understand the congenital malformations of the atlas, it is required to know its developmental process. The formation of the atlas starts from three ossification centers originating from the first sclerotome. Both lateral ossification centers begin growing posteriorly within 7 to 7 weeks of embryogenesis to form the posterior arch and unite against each other within 4 months.^32^ Atlas hypoplasia could be caused by a failure in the dorsal expansion of the two lateral primary ossification centers because of a chondrogenesis error or resulting from premature fusion or early ossification of the neural arches.^21,23^

The dimensions of the upper cervical canal were first studied by Steel^33^ in 1968, who proposed the “Rule of Thirds,” explaining why cervical stenosis at the atlas level was so rare. More recently, Kelly et al^2^ did a cadaveric study and established that the normal C1 inner sagittal diameter, measured from the most posterior aspect of the fovea dentis to the most posterior portion of the ventral lamina, ranged from 23.5 to 38.1 mm, with a mean of 30.8 ± 2.4 mm. Hypoplasia of the atlas was defined when the inner sagittal diameter of the atlas is less than 28.9 mm. This study defined an objective criterion for atlas hypoplasia in adults. In our case, this distance measured in CT scan was 23.3 mm in the axial plane and 23.5 mm in the sagittal plane (Figures 3 A and C). The normal sagittal diameter of the canal at this level, also known as PADI or SAC (space available for the cord), is 19 to 32 mm at C1^15^ measured from the posterior aspect of the dens until the anterior aspect of the posterior arch of the atlas. In our case, the PADI was 11 mm (Figures 3B and D), which is larger than the mean PADI obtained from this series, 8.8 mm. The sagittal diameter of the dural sac, measured from the transverse ligament to the posterior arch, is between 12 and 21 mm at C1^28^; in our case, it was reduced to 6.5 mm because of a hypertrophic transverse ligament.

### Clinical Presentation

**Figure 5:**
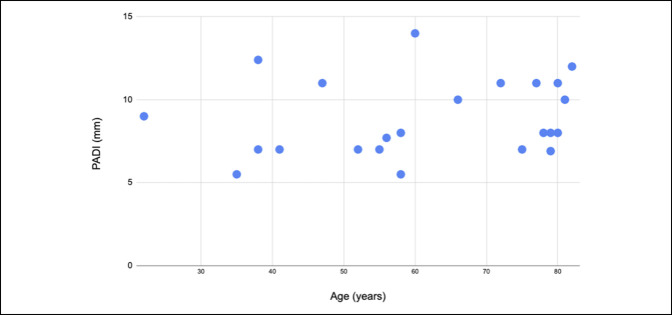
Correlation analysis between posterior atlanto-dens interval and patients' age. The correlation coefficient was of 0.19 showing a slight yet positive correlation of these factors.

The clinical presentation of C1 stenosis is similar to subaxial cervical myelopathy. Cervical pain, spasticity, gait and sensory disturbance, loss of manual dexterity, and urinary incontinence are the most often described symptoms.^23^ Trauma or degenerative changes^25^ such as ligamentum flavum buckling, pannus or synovial cyst formation, and ossification of the transverse ligament^7,26,27^ may act as stressors that exacerbate the symptomatology in elders. In our analysis, we found a slight yet positive correlation between PADI values and age of clinical presentation (Figure 5).

### Diagnosis

The diagnosis can be made with cervical lateral radiographs, CT scan, or cervical magnetic resonance imaging. The C2/C3 spinolaminar line test, proposed by Oshima et al,^30^ using a lateral cervical plain radiograph is a useful, sensitive (100%), and specific (97%) tool for screening. The prevalence of positive spinolaminar line in asymptomatic patients was 4.4%,^30,34^ 92.8% of them were older than 60 years old.^34^ The most accurate and specific diagnostic method was described by Kelly et al,^2^ when measuring an AP C1 canal diameter of less than 28.9 mm.

### Treatment

C1 stenosis treatment consists of a posterior atlas laminectomy.^3,6^ However, it is necessary to perform a thorough evaluation of the cervical spine and the craniovertebral junction. When atlantoaxial or craniocervical junction instability is found, concomitant fusion is necessary. Many patients underwent extended decompressions besides C1 laminectomy, and half of them do not report the follow-up period. As these procedures can cause instability, a longer follow-up should be required to determine the real outcomes of these decompressions.

### Limitations

We acknowledge that this study has its limitations. First of all, we conducted a literature review without applying a Preferred Reporting Items for Systematic Reviews and Meta-Analysis workflow. Although we could have missed some reports, we have included all the cases found by the time, applying strict criteria in each documented patient and obtaining all the relevant information. Second, our review is based on previous case reports and small series analysis, generating a low level of evidence and possible bias of patient selection and data collection. Finally, although we cannot generalize our statement as the level of evidence achieved is low, we are able to offer a complete guide for diagnosing, evaluating, and treating this pathology all over the world because we have confirmed that it is not a specific pathology of eastern population.

## Conclusion

Isolated fully formed C1 hypoplasia causing high cervical stenosis and myelopathy is an infrequent entity. It can present clinically at a wide range of ages and be successfully treated with C1 posterior arch excision alone if no further instability is detected. To our knowledge, this is the first case reported in America. We encourage surgeons of all over the world to consider this entity when C1 stenosis is present since it is not an exclusive anomaly of the Far East population.
